# Longitudinal Extension of the Win Odds for Ordinal Repeated Measurements

**DOI:** 10.1002/sim.70536

**Published:** 2026-04-10

**Authors:** Yongxi Long, Bart C. Jacobs, Ewout W. Steyerberg, Erik W. van Zwet

**Affiliations:** ^1^ Biomedical Data Sciences Leiden University Medical Center Leiden the Netherlands; ^2^ Department of Neurology Erasmus Medical Center Rotterdam the Netherlands; ^3^ Department of Immunology Erasmus Medical Center Rotterdam the Netherlands; ^4^ Julius Center for Health Sciences and Primary Care University Medical Center Utrecht Utrecht the Netherlands

**Keywords:** clinical trials, ordinal longitudinal outcomes, probabilistic index model, win odds

## Abstract

Initially proposed for analyzing composite endpoints, the win odds have recently received increasing interest for the analysis of ordinal outcomes. When comparing an ordinal outcome between two groups, the win odds are the odds that a randomly selected subject from the first group has a better outcome than a randomly selected subject from the second group. As such, the win odds are an effect size that is closely related to the Mann–Whitney U test. The win odds can be adjusted for covariates by the probabilistic index model. Here, we aim to extend this model for repeated measurements. We modify the estimation equations of the probabilistic index model to account for within‐subject correlation. Parameter estimation can be conveniently done via some data re‐structuring and the R package geepack. We implement a sandwich‐type estimator to estimate the variance‐covariance matrix. Simulations show that the estimation of the win odds is consistent and the coverage of confidence intervals is close to nominal. We provide an application by reanalyzing a neurological trial for the treatment of Guillain–Barré syndrome (SID‐GBS trial). This extension establishes the win odds as a promising summary measure to compare longitudinal ordinal outcomes. R package lwo is available on GitHub for implementing the proposed method.

**Abbreviations**
GBS: Guillain–Barré syndrome
PIM: probabilistic index model
RCT: randomized controlled trial

## 1 Introduction

Ordinal data are common in many scientific disciplines. For example, in psychometrics, the well‐known Likert scale is often used to measure opinions, attitudes, and motivations. In neurology, ordinal scales are frequently used to assess the health functioning of patients with neurological diseases, e.g., the modified Rankin Scale (mRS) [1] and the Glasgow Outcome Scale Extended (GOS‐E) [2].

If we assign numerical values to the categories of the ordinal scale, we can use statistical methods for numerical data, such as the t‐test and linear regression. Of course, this approach is only appropriate to the extent that the numerical values are a meaningful representation of the categories [3]. Ordinal scales are also often dichotomized so that statistical methods for binary data, such as logistic regression, can be used. Dichotomization is inefficient because it results in a considerable loss of information [4, 5]. It is statistically more efficient and rigorous to use methods that specifically address the discrete, ordered nature of ordinal outcomes. One such method is the proportional odds regression model [6], which is sometimes called a shift analysis. The treatment effect is then quantified as the “common odds ratio” which is interpreted as a general shift to higher (or lower) ordinal categories [7].

Another common choice for the comparison of ordinal outcomes is the rank‐based Mann–Whitney U test [8, 9]. However, it is not so clear what the relevant effect size is. The “probabilistic index” has been suggested as an appropriate choice [10]. Every subject in one group is compared to every subject in the other group to assess who has a better outcome, with ties broken by a coin toss. The probabilistic index is then the probability of a “win” of a subject from the first group over a subject from the second group. The associated odds are referred to as the “win odds” [11]. The straightforward interpretation of these “win statistics” has resulted in their considerable popularity in several fields, including cardiology and neurology [12, 13]. Moreover, a regression framework has been developed to model the effects of covariates on the probabilistic index [14]. This is called the Probabilistic Index Model. It is implemented in the R package pim [15].

Subjects may transition between ordinal categories over time. Studies with a longitudinal design can capture such varying trajectories and characterize changes in the effects of interest [16, 17]. Longitudinal data contain richer information than a single‐time measurement, potentially enhancing the statistical efficiency [18, 19, 20]. Unfortunately, longitudinal information remains underused despite the fact that many studies did have repeated measurements. A review of randomized controlled trials (RCTs) published in top medical journals revealed that while 79% of the RCTs had repeated outcome measurements, only 23% used all outcome data in their primary analyses [21]. Specifically, for ordinal longitudinal data, analyses rarely used both the ordering information and the repeated measurements [22, 23]. Often, either a single‐time‐point analysis was performed on the full ordinal scale, or a longitudinal analysis was done on the dichotomized ordinal scale [23].

Several extensions of the proportional odds model to longitudinal outcomes have been proposed. Within‐subject correlation may be modeled by adding random effects [24] or by using previous outcomes as predictors for the next outcome [22]. Here, our goal is to extend the Probabilistic Index Model to handle repeated measurements.

As a motivating example, we use data from the SID‐GBS trial [25]: A randomized controlled trial that investigated the effect of a second intravenous immunoglobulin dose in patients with severe Guillain–Barré syndrome (GBS). GBS is an acute, immune‐mediated disorder of the peripheral nerves characterized by a monophasic disease course [26]. Patients typically experience a rapid neurological deterioration, followed by a stabilization phase and a slow, often incomplete, recovery. The timing of disease progression varies substantially across individuals, ranging from a few days to several weeks. This heterogeneity leaves room for potential gains through longitudinal analysis.

The remainder of the paper is structured as follows. In Section 2, we provide the statistical background for the win statistics and provide a brief introduction to the Probabilistic Index Model (PIM). In Section 3, we propose our extension of the PIM to the longitudinal setting. We present its operating characteristics in a simulation study in Section 4. In Section 5, we demonstrate the method by re‐analyzing the SID‐GBS trial. We end with a brief discussion. An R package lwo has been developed to implement the proposed method (available at https://github.com/Yongxi‐Long/lwo). All code used to reproduce the simulation study and the SID‐GBS trial analysis is available at https://github.com/Yongxi‐Long/Longitudinal_Win_Odds, and is also included in the Appendix.

## 2 Statistical Background

The Mann–Whitney U (MWU) test is used to assess whether one distribution tends to yield larger values than the other [8]. Unlike t‐tests, the rank‐based MW test relies only on the ranks, so that it is well‐suited for the comparison of ordinal data [9]. Suppose we compare two groups of sizes $n_{1}$ and $n_{2}$ with outcomes X and Y. The MWU statistic is defined as the number of times that an X value is larger than a Y value. In the presence of ties, which is often the case for ordinal data, the MWU statistic is modified by assigning 1/2 for tied pairs [27].

$$\text{MWU}=\overset{n_{1}}{\underset{i=1}{\sum }}\overset{n_{2}}{\underset{j=1}{\sum }}\left(1\{X_{i}>Y_{j}\}+\frac{1}{2}1\{X_{i}=Y_{j}\}\right)$$

The MWU statistic may be standardized and compared to the standard normal distribution. Now, let B have the Bernoulli distribution with probability 1/2 (a “coin toss”) and define the event

X≽Y:={X>Y}∪[{X=Y}∩{B=1}]

The Probabilistic Index (PI) is the probability of this event

$$\text{PI}:=P(X≽Y)=P(X>Y)+\frac{1}{2}P(X=Y)$$

It is easy to see that $\text{MWU}/(n_{1}n_{2})$ is an unbiased estimator of the PI [28]. Therefore, the PI may be viewed as the effect size associated with the MW test [10, 29]. The null hypothesis of the MWU test is that the two distributions are equal, which immediately implies that PI = 1/2, but the converse does not necessarily hold. So the hypothesis that PI = 1/2 is more general than equality of distributions [11, 28].

The win odds are the odds of the PI, defined as PI/(1‐PI) [11, 30]. Other proposed “win statistics” [31] can also be derived from pairwise comparisons: The win ratio, which is the ratio of win proportions, and the net benefit, which is the difference in win proportions [32, 33]. Both these measures discard the ties. Here, we focus on the PI and win odds to retain the information contained in ties.

**FIGURE 1:**
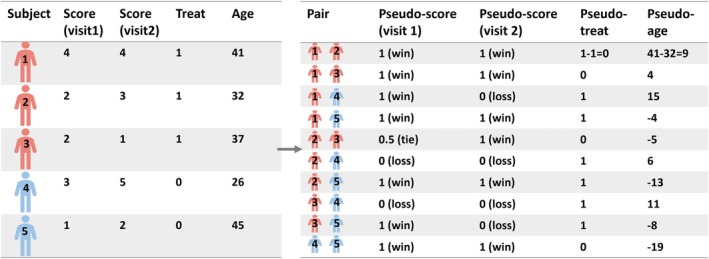
Formulation of ten pairs from a hypothetical cohort of five subjects at two visits. The left table presents individual‐level data, and the right table presents pseudo‐outcomes from pairwise comparisons and pseudo‐covariate values as the differences between individual covariate values.

When comparing two groups, the PI can be estimated as the proportion of wins plus half of the proportion of ties. However, it is not clear how to account for the effect of covariates. The Probabilistic Index Model (PIM) proposed by Thas et al. [14] allows flexible covariate adjustment by embedding the PI in a regression framework. Now, let Y be the ordinal outcome of interest and $X\in {\mathbb{R}}^{p}$ be a p‐dimensional vector of relevant baseline covariates. Consider a sample of n individuals with observations $\left(Y_{1},X_{1}),\ldots ,(Y_{n},X_{n}\right)$. In the simple two‐group comparison, we only need to consider the pairs that are formed by a subject from the first group and a subject from the second group. In the more general set‐up, we need to consider all possible N=n2 pairs (i,j) (Figure 1, we may freely choose some ordering). We define the events with the previous Bernoulli random variable B

$$Y_{i}≽Y_{j}:=\{Y_{i}>Y_{j}\}\cup \left[\left\{Y_{i}=Y_{j}\}\cap \{B_{ij}=1\right\}\right]$$

so that

$$P(Y_{i}≽Y_{j}|X_{i},X_{j})=P(Y_{i}>Y_{j}|X_{i},X_{j})+\frac{1}{2}P(Y_{i}=Y_{j}|X_{i},X_{j}).$$

We also define the indicator $I_{ij}=1\{Y_{i}≽Y_{j}\}$ so that $P(Y_{i}≽Y_{j}|X_{i},X_{j})=𝔼(I_{ij}|X_{i},X_{j})$.

Following the authors of PIM [14], we make the strong, but practical assumption that $I_{ij}$ depends on $\left(X_{i},X_{j}\right)$ only through the difference $Z_{ij}=X_{i}-X_{j}$. That is, we assume $𝔼(I_{ij}|X_{i},X_{j})=𝔼(I_{ij}|Z_{ij})$. To arrive at the probabilistic index model (PIM), we make one further assumption that $𝔼(I_{ij}|X_{i},X_{j})$ has the specific form

(1) $$𝔼(I_{ij}|X_{i},X_{j})=\text{expit}(\beta^{\prime }Z_{ij})$$

where β is a p‐dimensional vector of regression parameters. By using a logit link, we are modeling the log odds of the probabilistic index, which is the log of win odds. It is a logistic regression model applied to the transformed pair‐level pseudo‐outcomes $I_{ij}$ and pseudo‐covariates $Z_{ij}$. Of course, other link functions are also possible [14]. Note that the PIM has no intercept, which is a consequence of the fact that we have only pair‐level comparisons.

Thas et al. [14] proposed to estimate β by solving the following estimation equations

(2) $$U(\beta )=\underset{\left(i,j\right)}{\sum }U_{ij}(\beta )=\underset{\left(i,j\right)}{\sum }\frac{\partial \mu_{ij}}{\partial \beta^{\prime }}V_{ij}^{-1}(I_{ij}-\mu_{ij})=0$$

where $\mu_{ij}=𝔼(I_{ij}|Z_{ij})$. For simplicity, we use the variance function as if there were no ties [34], i.e., $V_{ij}=\mu_{ij}(1-\mu_{ij})$.

Even if the individual‐level data are independent, the pair‐level data are dependent. For example, the pseudo‐outcomes $I_{12}$ and $I_{13}$ are dependent because they share subject 1. To account for this dependence, a sandwich estimator is proposed for the variance‐covariance matrix [14]

(3) $$\begin{array}{ll} \hat{\text{Cov}}(\hat{\beta )} & ={\left\{\underset{\left(i,j\right)}{\sum }\frac{\partial U_{ij}(\hat{\beta })}{\partial \beta^{\prime }}\right\}}^{-1}\left\{\underset{\left(i,j\right)}{\sum }\;\underset{\left(k,l\right)}{\sum }\phi_{ijkl}U_{ij}(\hat{\beta })U_{kl}^{\prime }(\hat{\beta })\right\}\\ & \;\times {\left\{\underset{\left(i,j\right)}{\sum }\frac{\partial U_{ij}(\hat{\beta })}{\partial \beta^{\prime }}\right\}}^{-1} \end{array}$$

where

$$\phi_{ijkl}=\left\{\begin{array}{ll} 1,\; & \text{if}\;i=k\;\text{or}\;j=l\\ 0,\; & \text{otherwise} \end{array}\right.$$

The PIM is implemented in the R package pim [15].

Obtaining win odds from the PIM in Equation (1) is rather straightforward. As an example, consider an RCT. Let T denote the treatment assignment where T=1 is the experimental treatment and T=0 the control condition. Let X denote the patients' age, or some other prognostic factor to be adjusted for. The following PIM

$$\begin{array}{ll} P(Y_{i}≽Y_{j}|T_{i},T_{j},X_{i},X_{i}) & =𝔼(I_{ij}|T_{i},T_{j},X_{i},X_{i})\\ & =\text{expit}\left(\beta_{1}(T_{i}-T_{j})+\beta_{2}(X_{i}-X_{j})\right) \end{array}$$

gives the win odds of the treatment as $\exp({\hat{\beta }}_{1})$, adjusted for age.

## 3 Longitudinal Extension of the PIM

Suppose now we have a longitudinal study. The ordinal outcome of interest $Y_{i}^{t}$ is measured at visits t=1,…,T for individuals i=1,…,n. Let $X_{i}^{t}$ be a p×1 vector of covariates for the i‐th individual at visit t. It can include both time‐fixed baseline covariates and time‐dependent covariates, such as biomarker values. We now form pairs within each visit with pseudo‐outcome $I_{ij}^{t}$ and pseudo‐covariates $Z_{ij}^{t}=X_{i}^{t}-X_{j}^{t}$ (Figure 1). We may not necessarily have n2 pseudo‐pairs at each visit due to unbalanced data. The PIM in Equation (1) can be extended to the longitudinal setting by modeling the mean of the pseudo‐outcome for all visits

(4) $$𝔼(I_{ij}^{t}|X_{i}^{t},X_{j}^{t})=\text{expit}\left\{\beta^{\prime }Z_{ij}^{t}\right\}$$

We now have to account for the correlation between the same pair across different visits. We modify the estimation equations in Equation (2) by substituting $V_{ij}$ with $\sum_{ij}$

(5) $$U(\beta )=\underset{(i,j)\in {ℐ}_{N}}{\sum }U_{ij}(\beta )=\underset{(i,j)\in {ℐ}_{N}}{\sum }\frac{\partial \mu_{ij}}{\partial \beta^{\prime }}\sum_{ij}^{-1}(I_{ij}-\mu_{ij})=0$$

where $I_{ij}={\left(I_{ij}^{1},\ldots ,I_{ij}^{T}\right)}^{\prime }$, $\mu_{ij}=\mu_{ij}^{1},\ldots ,\mu_{ij}^{T}$ and $\sum_{ij}=V_{ij}^{1/2}R_{ij}V_{ij}^{1/2}$. $R_{ij}$ is a working correlation matrix [35] for repeated outcome measures on the same pair (i,j). $V_{ij}$ is now an T×T diagonal matrix with $\mu_{ij}^{t}(1-\mu_{ij}^{t})$ as the t‐th element of the diagonal.

If the pairs were independent, the covariance matrix would be readily estimated by the regular robust sandwich estimator [36]. But the pairs are sparsely correlated in the sense that some are independent while others are dependent due to sharing the same subject. The standard errors estimated from the regular sandwich estimator will be too small.

The pairs in a longitudinal setting have three different types of correlations. First, there is a within‐subject correlation due to repeated measurements on the same subjects. So the measurements of the pair (1,2) at different visits are correlated (Figure 1). Second, there is a correlation due to sharing the same patient. So pair (1,2) and pair (1,3) at the same visit are correlated. Thirdly, pairs (1, 2) and (1,3) at different visits are also correlated as a result of the two types of correlations.

To address these dependencies, we proceed in two steps. First, in the estimating equation (5), we added a working covariance matrix $\sum_{ij}$ with a specified working correlation matrix $R_{ij}$ to account for the temporal correlation of repeated measurements in parameter estimation. Second, for variance estimation, the usual “robust sandwich” estimator would suffice if pairs were independent. In that case, the “meat” of the sandwich would simply be the sum of cross‐products of the cluster score contributions, i.e., $\sum_{\left(i,j\right)}U_{ij}(\hat{\beta })U_{ij}^{\prime }(\hat{\beta })$.

However, our setting featured a mix of different types of correlation. We modified the “meat” part of the sandwich estimator from Equation (3) by including additional cross‐products of the correlated cluster score functions. This led to the following equation.

(6) $$\begin{array}{ll} \hat{\text{Cov}}(\hat{\beta )} & ={\left\{\underset{\left(i,j\right)}{\sum }\frac{\partial U_{ij}(\hat{\beta })}{\partial \beta^{\prime }}\right\}}^{-1}\left\{\underset{\left(i,j\right)}{\sum }\;\underset{\left(k,l\right)}{\sum }\phi_{\left(ijklst\right)}U_{ij}(\hat{\beta })U_{kl}^{\prime }(\hat{\beta })\right\}\\ & \;\times {\left\{\underset{\left(i,j\right)}{\sum }\frac{\partial U_{ij}(\hat{\beta })}{\partial \beta^{\prime }}\right\}}^{-1} \end{array}$$

We do not distinguish the three different types of correlation; instead, we simply use $\phi_{\left(ijklst\right)}$ to indicate whether pair (i,j) at time t is correlated with pair (k,l) at time s. Specifically,

$$\phi_{\left(ijklst\right)}=\left\{\begin{array}{ll} 1,\; & \text{if}\;i=k\;\text{or}\;j=l\\ 0,\; & \text{otherwise} \end{array}\right.$$

## 4 Simulation

We set up our simulation study in the context of the SID‐GBS trial [25]. The primary outcome was Guillain–Barré syndrome disability scale (GBS‐DS) at 4 weeks after the start of the standard intravenous immunoglobulin treatment. GBS‐DS is a seven‐category ordinal scale ranging from score 0 to score 6 (Table 1). GBS‐DS was also measured at week 1, 2, 8, 12, and 26. We consider covariate adjustment for age (continuous) and preceding diarrhea (binary, yes/no).

**TABLE 1:** Description of the Guillain–Barré syndrome disability scale (GBS‐DS).

Score	Description
0	completely normal
1	mild symptoms or signs, but able to run
2	can walk independently ≥10 meters without help, but cannot run
3	can walk 10 m with help
4	bedridden or requiring wheelchair
5	need assisted ventilation
6	death

Our estimand of interest is the log win odds at an arbitrary time point t after baseline, adjusted for age and preceding diarrhea, denoted as $\theta^{t}$.

$$\theta^{t}=\log\left\{\frac{P(Y_{\text{treat}}^{t}≽Y_{\text{control}}^{t}|\text{age, preceding diarrhea})}{P(Y_{\text{control}}^{t}≽Y_{\text{treat}}^{t}|\text{age, preceding diarrhea})}\right\}$$

We assessed the performance of the estimator ${\hat{\theta }}^{t}$ from the longitudinal probabilistic index model (4) using the following metrics: (i) Bias. (ii) Difference of the model variance from the sandwich estimator compared to the Monte Carlo variance. (iii) Coverage of the 95% confidence intervals. For each scenario, we ran 10 000 iterations to ensure that the Monte Carlo standard error of the coverage probability was approximately $\sqrt{0.95(1-0.95)/10000}=0.002$.

### 4.1 Data Generating Process

We used GBS‐DS as our outcome and considered two visits after baseline (week 0), week 4, and week 8. We lumped the seven‐category GBS‐DS into five categories (combined score 0 with score 1 and score 6 with score 5) because score 0 and score 6 were not observed for the primary outcome of the SID‐GBS trial.

The ordinal outcome GBS‐DS at each time point was generated from a proportional odds model as follows

$$\begin{array}{ll} P({\text{GBS‐DS}}_{i}^{t}\leq k)= & \text{expit}\left(\beta_{0k}+\beta_{1}{\text{week4}}_{i}+\beta_{2}{\text{week8}}_{i}\right.\\ & \;+\beta_{3}{\text{treat}}_{i}\cdot {\text{week4}}_{i}+\beta_{4}{\text{treat}}_{i}\cdot {\text{week8}}_{i}\\ & \;\left.+\beta_{5}{\text{age}}_{i}+\beta_{6}{\text{preceding diarrhea}}_{i}\right) \end{array}$$

$\beta_{1},\beta_{2}$ are the time effects, and $\beta_{3},\beta_{4}$ are the treatment effects at weeks 4 and 8, respectively. $\beta_{5}$ and $\beta_{6}$ are the prognostic effects of age and preceding diarrhea. To obtain correlated GBS‐DS measurements on the same patient, we used the genOrdCat function from the simstudy package [37]. It generates correlated values from the logistic distribution using a standard normal copula‐like approach with a supplied correlation matrix.

We took our covariate distribution from the SID‐GBS trial. Age was generated from a normal distribution with a mean of 60 and a standard deviation of 10. The status of preceding diarrhea was generated from a Bernoulli distribution with a mean of 0.4.

### 4.2 Simulated Scenarios

We considered four different scenarios (Table 2). The first scenario is the null scenario of no treatment effect throughout follow‐up. The second scenario is a mimic of the neural SID‐GBS trial. The third is a trial with increasing treatment effect over time, and the fourth is a trial with constant treatment effect over time. The correlation matrix is the autoregressive of order one $R=\left[\begin{array}{lll} 1 & \rho & \rho^{2}\\ \rho & 1 & \rho \\ \rho^{2} & \rho & 1 \end{array}\right]$. We varied the autocorrelation coefficient ρ to achieve different strengths of within‐subject correlation. For each scenario, we assessed the influence of varying sample sizes. The intercepts $\beta_{0k}$'s were set to −3.8,−2.2,−1.3,0.7 to match the marginal proportion of GBS‐DS categories observed in the SID‐GBS trial.

**TABLE 2:** Design parameter values for simulation scenarios.

Scenario	Description	Sample size	ρ	$\beta_{1}$	$\beta_{2}$	$\beta_{3}$	$\beta_{4}$	$\beta_{5}$	$\beta_{6}$	Win odds (week 4)	Win odds (week 8)
1	Null	50, 100, 200	0.3, 0.6	0.6	1.2	0	0	−0.005	0.23	1	1
2	SID‐GBS trial mimic	50, 100, 200	0.3, 0.6	0.6	1.2	−0.08	−0.16	−0.005	0.23	0.95	0.91
3	Increasing treatment effect	50, 100, 200	0.3, 0.6	0.6	1.2	0.4	0.8	−0.005	0.23	1.27	1.65
4	Constant treatment effect	50, 100, 200	0.3, 0.6	0	0	1	1	−0.005	0.23	1.80	1.80

**FIGURE 2:**
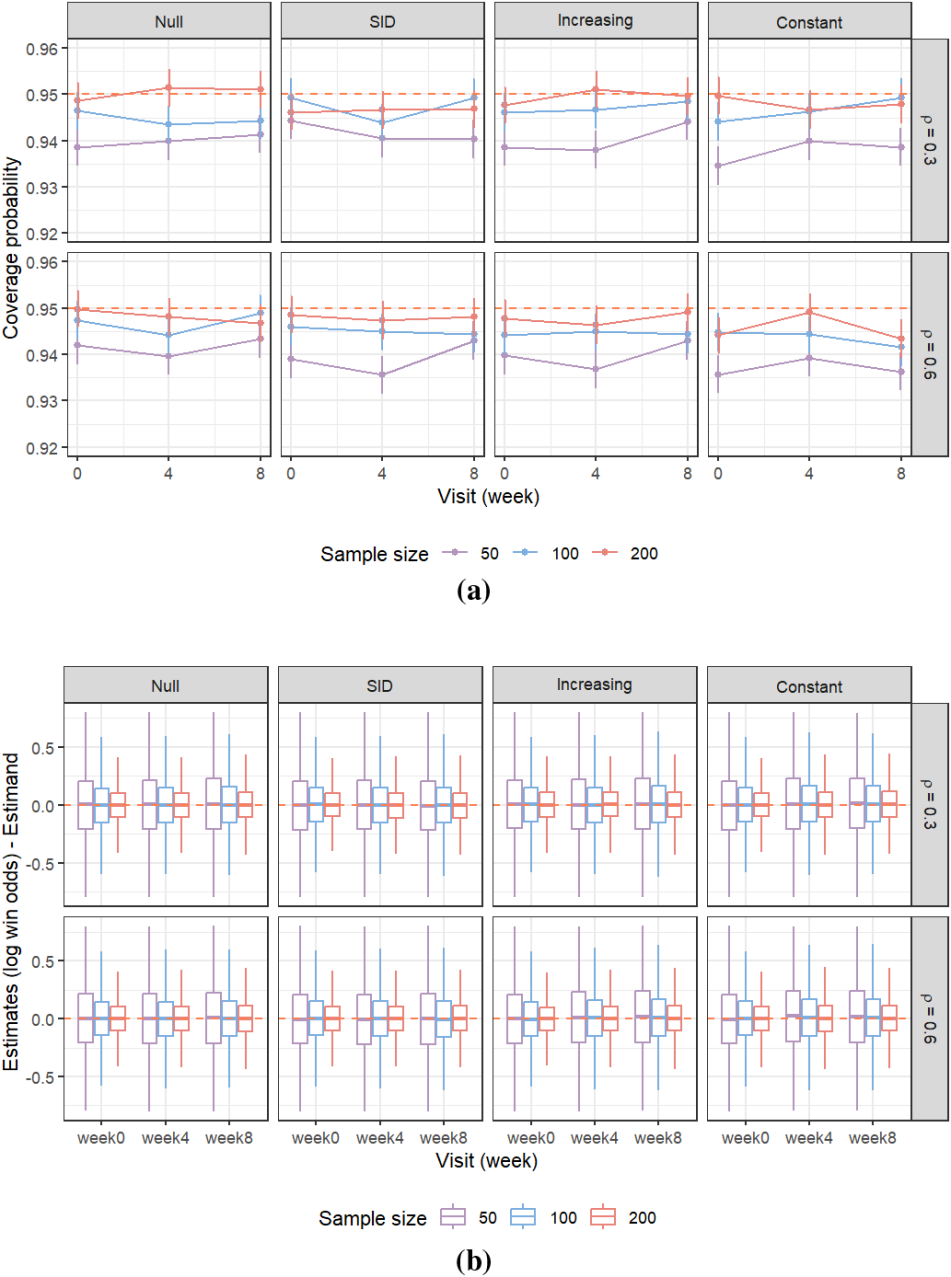
Simulation results. (a) Coverage probability of longitudinal win odds at week 0, 4, 8 for scenarios presented in Table 2. Each scenario has 10 000 Monte Carlo simulated data sets, resulting in a Monte Carlo standard error of about 0.002 for the simulated coverage probability of 0.95. (b) Boxplot of difference between estimates of log win odds (${\hat{\theta }}^{t}$) and estimands ($\theta^{t}$) at week t=0,4,8 for scenarios presented in Table 2.

The bias and the coverage probabilities along with the Monte Carlo 95% confidence interval for each simulation scenario are shown in Figure 2. The coverage probabilities are not systematically influenced by the magnitude of the win odds or the strength of the within‐subject correlation. The sandwich estimator tends to slightly underestimate the variance when the sample size is relatively small (n=50). The coverage probabilities converge to the target level as the sample size increases.

## 5 An Application to the SID‐GBS Trial

As an example, we performed a re‐analysis of the SID‐GBS trial [25] to illustrate our proposed method. In the SID‐GBS trial, the primary analysis of GBS‐DS at week 4 post‐randomization reported a common odds ratio of 1.4 (95% CI: 0.6–3.3) from the proportional odds model, adjusted for pre‐randomization covariates, including age, preceding diarrhea, and baseline MRC sum score. We used data from all six visits (week 1, 2, 4, 8, 12, and 26) after baseline and modeled the trajectory of the treatment effect during follow‐up.

**FIGURE 3:**
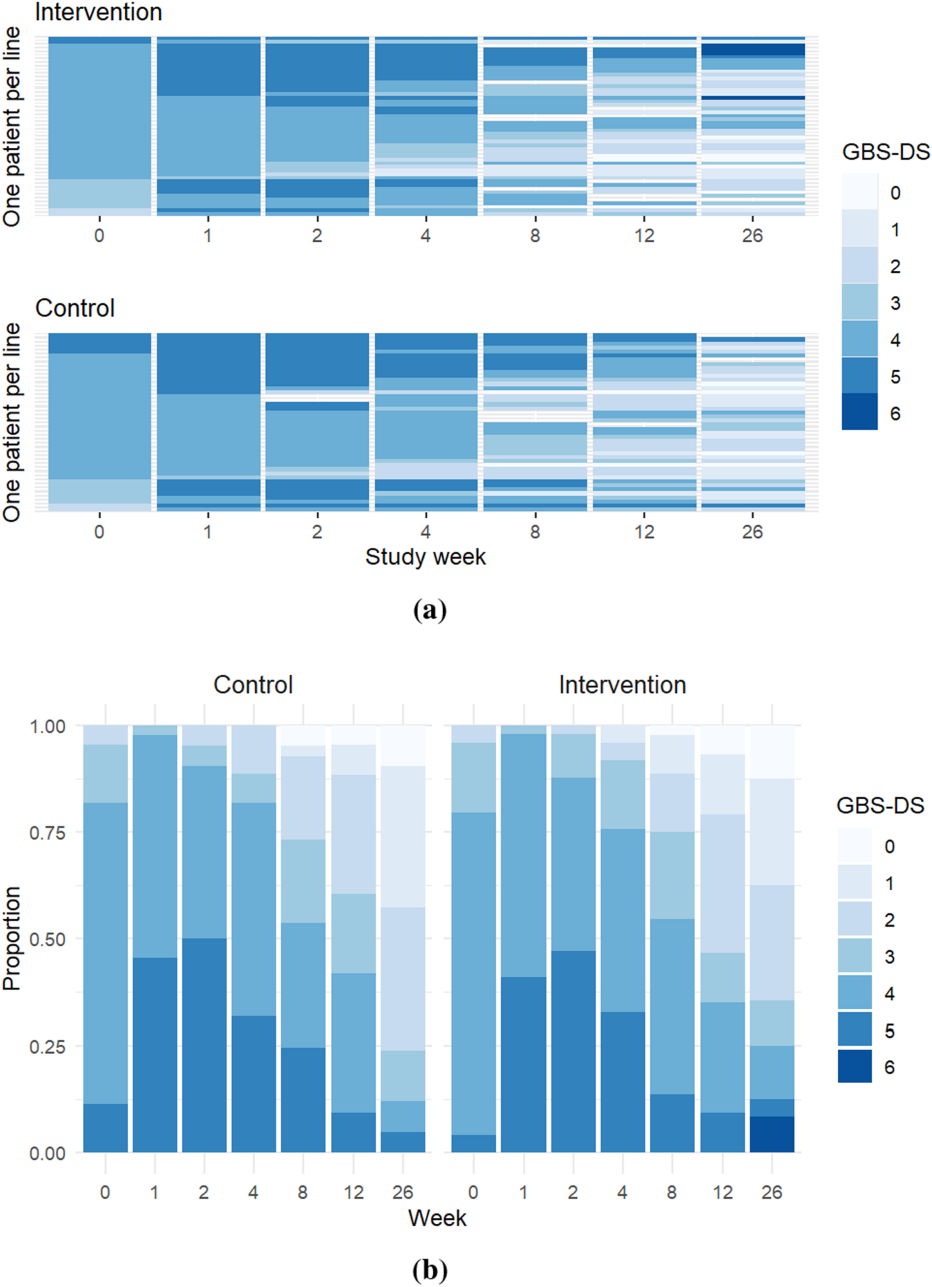
(a) Line chart of GBS‐DS status over the follow‐up in the SID‐GBS trial, each line represents one patient trajectory. (b) Stacked bar chart of observed proportions of GBS‐DS scores over the follow‐up in the SID‐GBS trial.

We visualized individual trajectories of GBS‐DS status in Figure 3. We can observe a general trend towards better (lower) GBS‐DS scores after an initial deterioration. This agrees with the natural history of GBS that symptoms peak within 4 weeks, followed by a recovery period for most patients [26]. The white gaps in trajectories indicate that some patients had missed outcome measurements at several visits (Figure 3a). The SID‐GBS trial aimed to evaluate the effect of a second dose of intravenous immunoglobulin in GBS patients with poor prognosis, so we can see that about 80% of patients were in relatively severe status (GBS‐DS categories 4 and 5) at baseline (Figure 3b). The proportion of patients being able to walk (GBS‐DS categories 0–3) at week 26 is almost 80%. The change of the outcome distribution over time is similar between the control and the intervention group.

**FIGURE 4:**
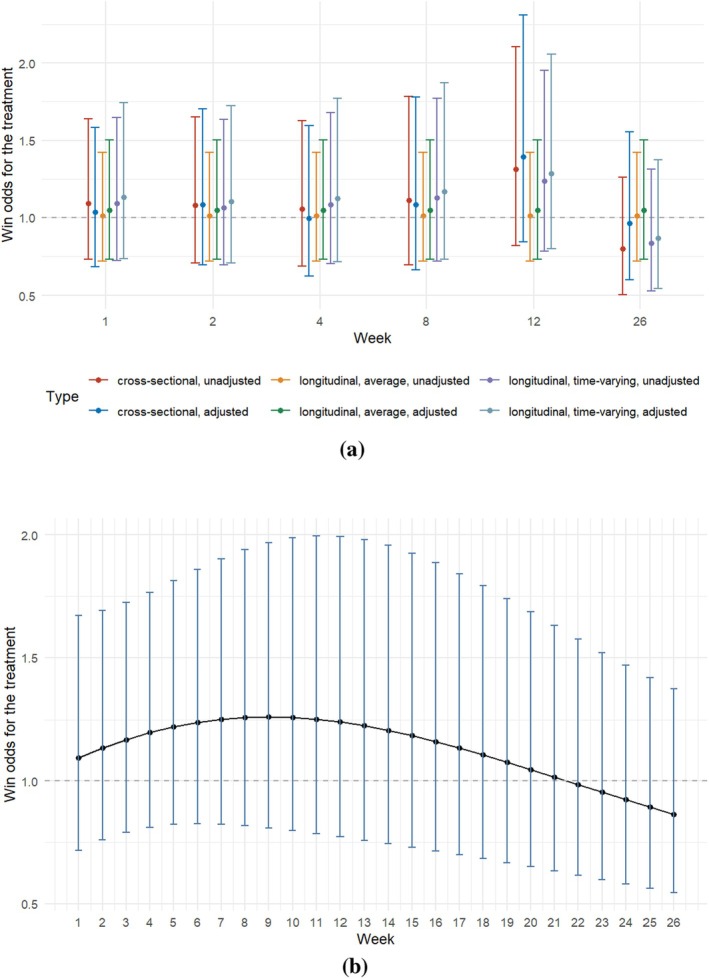
Estimated win odds with point‐wise 95% confidence intervals from different modeling approaches. (a) Cross‐sectional win odds; Longitudinal average win odds; Longitudinal time‐varying win odds. For each type, the unadjusted estimate is followed by the estimate adjusted for age, preceding diarrhea, and baseline GBS‐DS. (b) Spline with a knot at week 2 for the trajectory of win odds, adjusted for age, preceding diarrhea, and baseline GBS‐DS.

First, we fitted multiple single‐time PIMs to estimate the win odds separately for each visit. Then we applied the proposed longitudinal PIM (4) to simultaneously model all visits. We estimated the average win odds over time, as well as the time‐varying win odds where the time (as a categorical variable) interacted with the treatment. Figure 4a presents the three types of win odds, both unadjusted and adjusted for age, preceding diarrhea, and baseline GBS‐DS score. The estimates from different approaches are generally similar and indicate a neutral overall effect. The average win odds over time are close to one. The time‐varying win odds peak between week 4 and week 12, then start to wear off (Figure 4a).

Compared to the single‐time PIM, the longitudinal PIM provides a less noisy covariate adjustment, resulting in shorter confidence intervals. This is achieved by estimating a single set of baseline covariate effects across all visits. Additionally, the PIM quantifies the covariate effects in win odds as well, which should be interpreted in the context of pairwise comparisons. For instance, the estimate for the coefficient of preceding diarrhea is −0.04, which means that for two randomly selected patients with the same treatment assignment, age and baseline GBS‐DS score, evaluated at the same time point, the odds that the patient with preceding diarrhea has a better (lower) GBS‐DS score is estimated to be exp(−0.04)=0.96 (95% CI: 0.66–1.39), compared to the patient without preceding diarrhea.

We can also model the trend of the treatment effect over time in a continuous way. For example, we modeled the time‐by‐treatment interaction by natural cubic splines, with a knot at week 2 based on previous exploratory visualizations (Figure 4b). Throughout the follow‐up period, no significant treatment benefit of a second immunoglobulin dose over placebo was observed in patients with severe GBS.

## 6 Discussion

In this paper, we propose an extension of the probabilistic index model to analyze ordinal longitudinal outcomes. The treatment effect is quantified as the win odds, which can be interpreted as the odds of a randomly selected patient from the treatment group having a better outcome than a randomly selected control, with ties split evenly. The straightforward interpretation of the win odds has been argued to be more communicable than the common odds ratio from the proportional odds model [38, 39], which is a weighted average of binary odds ratios across all possible dichotomizations of the ordinal scale. However, note that the win odds are not the same as the causal effect, which is the odds that a randomly selected patient will have a better outcome under treatment than under control. This is not identifiable from randomized controlled trials [40]. By formulating the win odds within a regression framework, the longitudinal PIM offers greater flexibility in adjusting for covariates and handling clustered or repeated measurements, compared to previously proposed stratified win odds approaches [41] and Mann–Whitney‐type tests for clustered data [42].

In monophasic disorders such as the Guillain–Barré syndrome and traumatic brain injury, the timing of outcome assessment is a frequent topic of debate, mainly due to variability in disease progression and recovery trajectories among individuals [43, 44]. A single‐time point analysis can be either too early—before meaningful recovery has occurred, or too late—the treatment effect has waned, to demonstrate a difference. The proposed longitudinal PIM addresses this challenge by providing a more comprehensive assessment of the treatment benefit in time‐sensitive settings.

The PIM is essentially a logistic regression model estimating the conditional mean of the pseudo‐outcomes. Therefore, estimation can be done conveniently via existing software. We use the R package geepack with extra scripting to implement the sandwich‐type estimator for estimating the variance‐covariance matrix separately. We do not distinguish between the three types of correlations but instead assess whether pairs are correlated. It has been shown that subjects do not necessarily need to share the same correlation structure for the sandwich estimator to be consistent [36]. Consequently, the sandwich estimator remains robust even when the correlation structure of the pairs is misspecified. Slight underestimation of the variance is observed under a sample size of 50 due to the small sample bias of the sandwich estimator. The bootstrap can reduce the bias [45], but the coverage is already acceptable in our opinion. We tried Mancl and DeRouen's bias‐corrected robust variance estimator [46], but it did not meaningfully improve the coverage. Application of the proposed method to very small sample sizes is not recommended, due to bias [47] and potential separation issues [48] in logistic regression. Note that for a sample size of n subjects, a total of n(n−1)/2 comparisons are made at each visit. The computation time can become a burden with very large sample sizes [15].

The extension of the probabilistic index model is also useful for longitudinal continuous outcomes with outliers or skewed distributions. Because the probabilistic index only exploits ordering information, it is not affected by order‐preserving transformations of the outcomes. However, this robustness may come with the cost in terms of statistical efficiency, as the quantitative information contained in continuous outcomes is not used [28, 49]. Win odds have also received attention for analyzing composite time‐to‐event outcomes, in fields such as cardiology [13]. In that case, one should caution against its dependency on follow‐up time [31]. For instance, win odds may fail to show the long‐term benefit of a more important but less frequent category if the follow‐up time is not sufficient. Moreover, informative censoring can cause bias in win odds [50].

Via this work, we hope to add an additional tool to the analysis of longitudinal ordinal outcomes. More work is needed to compare the performance of the PIM extension to other longitudinal models for ordinal data, such as the proportional odds random effect model [51] and the Markov model [22]. Future research could explore methods for handling unbalanced longitudinal designs with irregular visit schedules, where aligning observations to form pseudo‐pairs at the same time can be challenging. Overall, we consider the extension of win odds as a promising summary measure to compare longitudinal ordinal outcomes.

## Funding

This work was supported by Annexon Biosciences.

## Conflicts of Interest

The authors declare no conflicts of interest.

## Supporting information


**Appendix S1:** sim70536‐sup‐0001‐AppendixS1.pdf.

**Appendix S2:** sim70536‐sup‐0002‐AppendixS2.pdf.

## Data Availability

The data that support the findings of this study are available on request from the corresponding author. The data are not publicly available due to privacy or ethical restrictions.
