# Supplementary material for: Longitudinal Extension of the Win Odds for Ordinal Repeated Measurements
Source: Stat Med. 2026 Apr 10;45:e70536. doi: 10.1002/sim.70536 (PMC13068635; doi:10.1002/sim.70536)
Supplement: Supplementary file 1 — Appendix S1: sim70536‐sup‐0001‐AppendixS1.pdf. [file SIM-45-0-s001.pdf]

Simulation study: Longitudinal extension of the win odds for ordinal repeated measurements

Yongxi Long

Table of contents

Aim . . . 1
1. Data generating process . . . 2
   Global parameters (fixed through-out) . . . 2
   Scenario-specific parameters . . . 3
   Estimands at each visit by construction . . . 4
2. Perform the simulation across 24 scenarios . . . 5
3. Results assessment . . . 6
plot of spread of estimates (log win odds) . . . 6
plot of coverage for all scenarios . . . 8

Aim

The script sets up the simulation scenarios and evaluates the simulation results.

The package can be installed from GitHub via

```
remove.packages("lwo")
# install.packages("pak")
pak::pak("Yongxi-Long/lwo")
```

```
rm(list=ls())
suppressPackageStartupMessages(
  {
library(lwo)
```

```r
library(dplyr)
library(ggplot2)
library(latex2exp)
  }
)
# self-defined function to evaluate simulation results
source("assess_results.R")
```

1. Data generating process

We elicit our simulation parameters from the SID-GBS trial. We used GBS-DS as our outcome and considered two visits after baseline (week 0), week 4 and week 8. We lumped the seven category GBS-DS into five-category (combined score 0 with score 1 and score 6 with score 5) because score 0 and score 6 were not observed for the primary outcome of the SID-GBS trial.

The ordinal outcome GBS-DS at each time point was generated from a proportional odds model as follows

$$
\begin{aligned}
P(GBS-DS_i^t \leq k) = \text{expit}\Big(&\beta_{0k} + \beta_1, \text{week4}_i + \beta_2, \text{week8}_i \\
&+ \beta_3, \text{treat}_i \cdot \text{week4}_i + \beta_4, \text{treat}_i \cdot \text{week8}_i \\
&+ \beta_5, \text{age}_i + \beta_6, \text{preceding diarrhea}_i\Big)
\end{aligned}
$$

Global parameters (fixed through-out)

```r
# number of iterations for each simulation scenario
nsim <- 1e4

# consider baseline, week 4 and week 8
visits <- c("week0","week4","week8") # baseline is visit = 0

# correlation structure for repeated measurements
corstr <- "ar1"

# the intercepts beta_0k's are determined by the base probabilites
# at time 0, resemble the SID-GBS trial
# Here GBS-DS category 01 and 56 were combined
# reversed such that higher score means better condition
```

```r
baseprobs <- rev(c(0.06,0.11,0.12,0.50,0.21))

# beta5 and beta6: covariate effects estimated from SID trial
# this is estimated from SID trial (after week 1)
covs_effects <- c("age"=-0.005,"pre_diarrhea"= 0.23)
```

Scenario-specific parameters

We evaluate four treatment effect patterns:

- a null scenario where there is no treatment effect, but there are covariate effects and main time effects (patients generally get better over time).
- a scenario that mimics the SID trial, the almost null treatment effect is derived from SID trial data
- a hypothetical positive SID trial where the treatment also interacts with time and therefore magnifies over time.
- a constant treatment effect

We also evaluate under three sample sizes ($N = 50, 100, 200$) and two correlation coefficients for data generation ($\rho = 0.3, 0.5$). This gives us in total 24 simulation scenarios.

```r
# four treatment effect patterns
scenarios <- c("null","SID","pos","con")

# three sample sizes
sample_sizes <- c(50,100,200)

# two correlation coefficients
rhos <- c(0.3,0.6)

# beta1 and beta2: main time effects
time_effects_lookup <- list(
  null = c(0.6, 1.2),
  SID  = c(0.6, 1.2),
  pos  = c(0.6, 1.2),
  con  = c(0, 0)
)

# beta3 and beta4: time-treatment interaction effects
time_trt_effects_lookup <- list(
  null = c(0, 0),
```

```r
  SID  = c(-0.08, -0.16),
  pos  = c(0.4, 0.8),
  con  = c(1, 1)
)

# all scenario combinations
grid <- expand.grid(
  scenario = scenarios,
  n = sample_sizes,
  rho = rhos,
  stringsAsFactors = FALSE
) |>
  arrange(scenario)

# build list of parameters
scenario_list <- apply(grid, 1, function(row) {
  list(
    scenario = row[["scenario"]],
    N = as.numeric(row[["n"]]),
    rho = as.numeric(row[["rho"]]),
    time_effects = time_effects_lookup[[row[["scenario"]]]],
    time_trt_effects = time_trt_effects_lookup[[row[["scenario"]]]]
  )
})
```

Estimands at each visit by construction

- PIM gives marginal effect (conditional on the difference of the covariate value between two subjects being zero instead of the absolute value of the covariate being zero).
- The PIM adjusted for age X_1 and preceding diarrhea X_2 is

$$P(Y_j^{(t)} \succcurlyeq Y_i^{(t)} | A_j = 1, A_i = 0, \mathbf{X_j}, \mathbf{X_i}) = g^{-1}\Big(\beta_0 + \beta_1 \text{visit}_1 + \beta_2 \text{visit}_2 \\ + \gamma_1 (X_{1j} - X_{1i}) + \gamma_2 (X_{2j} - X_{2i})\Big)$$

- $\beta_0, \beta_0 + \beta_1, \beta_0 + \beta_2$ is the adjusted log win odds at baseline, visit 1, and visit 2 respectively.
- It is not conditional on age being zero and preceding diarrhea being at the reference category, but on being of the same age and preceding diarrhea group. Still have to average over age & preceding diarrhea strata, like a marginal effect.

- To compute the true marginal win odds, generate a large data set and use stratification and weighting to approximate marginal win odds, the larger the N_approx, the more precise.

```
set.seed(86)
scenarios <- c("null","SID","pos","con")
scenario_effects <- lapply(scenarios, function(scen) {
  list(
    scenario = scen,
    time_effects = time_effects_lookup[[scen]],
    time_trt_effects = time_trt_effects_lookup[[scen]]
  )
})
names(scenario_effects) <- scenarios
# calculate estimand
true_wos <- lapply(scenario_effects, function(pars) {
  calculate_win_odds(
    N_approx = 1e6,
    baseprobs = baseprobs,
    covs_effects = covs_effects,
    time_effects = pars$time_effects,
    trt_ratio = 1,
    time_trt_effects = pars$time_trt_effects,
    visits = visits,
    corMatrix = diag(length(visits))
  )
})
save(true_wos,file = "estimands_win_odds.RData")
```

2. Perform the simulation across 24 scenarios

```
# load the estimand
load("estimands_win_odds.RData")
# loop through all scenarios
for(sce in scenario_list)
{
  scenario <- sce$scenario
  N <- sce$N
  rho <- sce$rho
  corMatrix <- gen_corMatrix(n_visits = length(visits),
```

```r
                                    rho = rho,
                                    corstr = corstr)
  time_effects <- sce$time_effects
  time_trt_effects <- sce$time_trt_effects
  estimand <- true_wos[[scenario]]
  source("LWO_simulation.R")
}
```

3. Results assessment

- Wald test for the global null hypothesis:

$$H_0 : \beta_0 = \beta_1 = \beta_2 = 0$$

plot of spread of estimates (log win odds)

```r
load("estimands_win_odds.RData")
bias_list <- sapply(scenario_list, function(sce)
{
  scenario <- sce$scenario
  N <- sce$N
  rho <- sce$rho
  estimand <- true_wos[[scenario]]
  load(paste0("results/results-comp-N",N,"-rho",rho,"-",scenario,".RData"))
  bias <- data.frame(t(sapply(results, function(i) return(i[[1]]-log(estimand)))))
  colnames(bias) <- paste0("week",c(0,4,8))
  out <-  reshape2::melt(bias,measure.vars=paste0("week",c(0,4,8)),
                         value.name = "bias",variable.name="visit")
  out$sample_size <- N
  out$rho <- rho
  out$scenario <- scenario
  return(out)
},simplify = FALSE)
bias_df <- dplyr::bind_rows(bias_list)

# bias_df |>
#   group_by(scenario,sample_size,rho,visit) |>
#   summarise(bias = mean(bias))
```

```r
bias_df$rho_f <- factor(bias_df$rho,
                             levels=c(0.3,0.6),
                             labels=c(TeX("$\\rho$ = 0.3"),TeX("$\\rho$ = 0.6")))
bias_df$scenario <- dplyr::case_when(
  bias_df$scenario == "null" ~"Null",
  bias_df$scenario == "pos" ~"Increasing",
  bias_df$scenario == "con" ~"Constant",
  bias_df$scenario == "SID" ~ "SID"
)
ggplot(bias_df,aes(x=visit,y=bias,color=factor(sample_size)))+
  facet_grid(rho_f~scenario,labeller = label_parsed)+
  geom_boxplot(outlier.shape = NA)+
  labs(color="Sample size"
       #,title = "Monte Carlo standard error = 0.002 (10,000 simulations)"
       )+
  theme_bw()+
  ylim(c(-0.8,0.8))+
  geom_hline(yintercept = 0,linetype=2,color="coral")+
  labs(x="Visit (week)",y="Estimates (log win odds) - Estimand")+
  scale_color_manual(values = c("#B395BD","#7DAEE0","#EA8379"))+
 # scale_x_continuous(breaks = c(0,4,8))+
  theme(
    legend.position = "bottom"
  )
```

```
Warning: Removed 4061 rows containing non-finite outside the scale range
(`stat_boxplot()`).
```

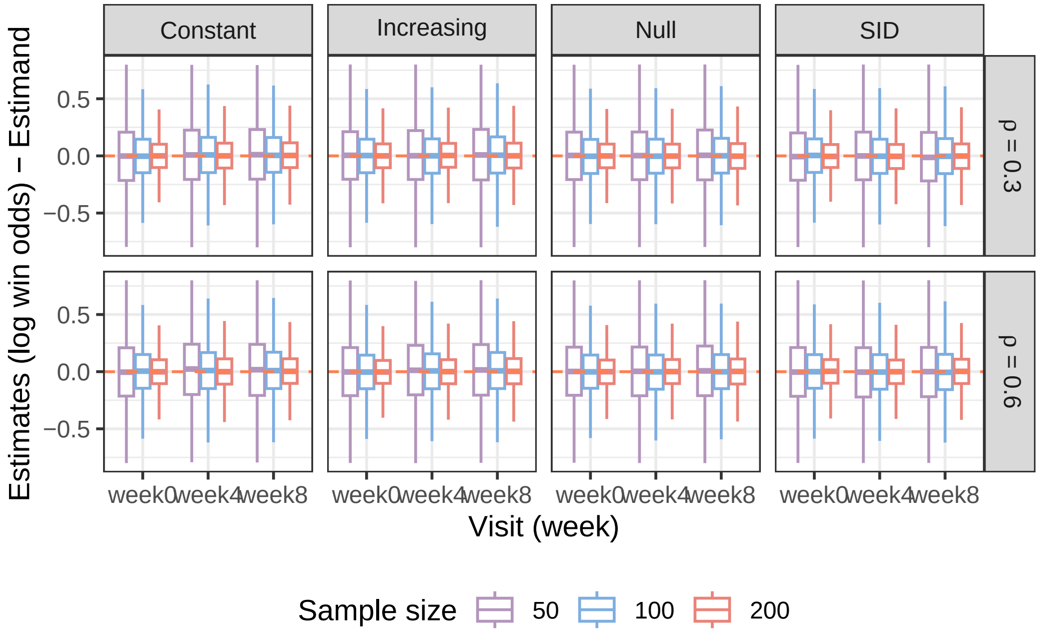

plot of coverage for all scenarios

```
coverage_list <- sapply(scenario_list, function(sce)
{
  scenario <- sce$scenario
  N <- sce$N
  rho <- sce$rho
  estimand <- true_wos[[scenario]]
  res <- assess_results(N=N,rho=rho,scenario = scenario,
                        estimand = estimand)
  coverage <- res$`table of performance metrics`$coverage
  out <- data.frame(
    scenario = scenario,
    N = N,
    rho = rho,
    visits=c(0,4,8),
    coverage = coverage
  )
  return(out)
},simplify = FALSE)
```

```r
coverage_df <- bind_rows(coverage_list)
coverage_df$sd <- 0.002
coverage_df$scenario <- dplyr::case_when(
  coverage_df$scenario == "null" ~"Null",
  coverage_df$scenario == "pos" ~"Increasing",
  coverage_df$scenario == "con" ~"Constant",
  coverage_df$scenario == "SID" ~ "SID"
)
coverage_df$rho_f <- factor(coverage_df$rho,
                             levels=c(0.3,0.6),
                             labels=c(TeX("$\\rho$ = 0.3"),TeX("$\\rho$ = 0.6")))
ggplot(coverage_df,aes(x=visits,y=coverage,group=factor(N),color=factor(N)))+
  facet_grid(rho_f~scenario,labeller = label_parsed)+
  geom_line()+
  geom_point()+
  labs(color="Sample size"
       #,title = "Monte Carlo standard error = 0.002 (10,000 simulations)"
       )+
  theme_bw()+
  ylim(c(0.92,0.96))+
   geom_errorbar(aes(ymin=coverage-1.96*sd, ymax=coverage+1.96*sd), width=.2,
                 position=position_dodge(0.05))+
  geom_hline(yintercept = 0.95,linetype=2,color="coral")+
  labs(x="Visit (week)",y="Coverage probability")+
  scale_color_manual(values = c("#B395BD","#7DAEE0","#EA8379"))+
  scale_x_continuous(breaks = c(0,4,8))+
  theme(
    legend.position = "bottom"
  )
```

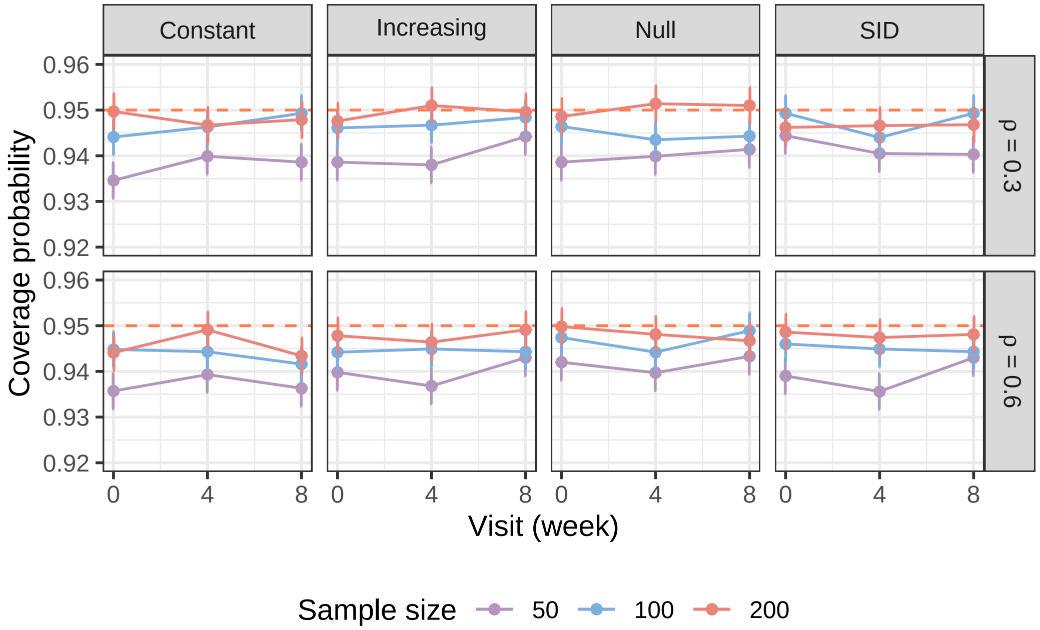
Constant
Increasing
Null
SID
ρ = 0.3
ρ = 0.6
Coverage probability
0.96
0.95
0.94
0.93
0.92
0
4
8
Visit (week)
Sample size
50
100
200
