# Supplementary material for: Longitudinal Extension of the Win Odds for Ordinal Repeated Measurements
Source: Stat Med. 2026 Apr 10;45:e70536. doi: 10.1002/sim.70536 (PMC13068635; doi:10.1002/sim.70536)

Analysis example using the longitudinal probabilistic index model

Yongxi Long

Table of contents

Aim . . . 1
Data . . . 2
  SID-GBS trial data . . . 2
  Simulated data set . . . 2
Exploratory analysis . . . 4
  Proportion of categories over time . . . 4
  Empirical cumulative probability of each category . . . 5
  Individual horizontal line plot . . . 7
Separate cross-sectional win odds analysis . . . 9
Longitudinal win odds analysis . . . 10
  Analysis 1: Average win odds over time . . . 10
  Analysis 2: Response profile modelling (time as a categorical covariate) . . . 12
  Result visualization . . . 14
Analysis 3: model treatment effect trajectory through spline . . . 16
  Result visualization . . . 17

Aim

This script is to demonstrate how to fit a longitudinal probabilistic index model using the *lwo* package and estimate adjusted win odds for each visit.

The package can be installed from GitHub via

```
remove.packages("lwo")
# install.packages("pak")
pak::pak("Yongxi-Long/lwo")
```

```
rm(list = ls())
suppressPackageStartupMessages(
  {
  library(lwo) # for fitting the longitudinal PIM
  library(ggplot2) # for plotting
  library(splines) # for the natural cubic splines function
  library(dplyr) # for data manipulation
  library(simstudy) # for longitudinal ordinal data generation
  library(ggpubr) # for arranging plots
  library(pim) # for single-time probabilistic index model
  library(fastDummies) # for making dummy variables
  }
)
# user-defined colors for plotting
blues <- c("#f7fbff", "#deebf7",
           "#c6dbef", "#9ecae1",
           "#6baed6", "#3182bd", "#08519c")
```

Data

SID-GBS trial data

The paper used SID trial patient data for illustration. The patient data is not publicly available. So for your own try-out, please run the code of the next section to get a simulated data set.

```
load(file = "SID_long_cleaned.RData")
load(file = "SID_wide_cleaned.RData")
dat_wide <- SID |>
  filter(!is.na(treat_ITT))
dat_long <- SID_long |>
  filter(!is.na(treat_ITT))
```

Simulated data set

We use the *lwo::gen_data()* function to simulate ordinal repeated measurement. The data generation is designed upon te SID trial (Walgaard, C., Second intravenous immunoglobulin dose in patients with Guillain-Barré syndrome with poor prognosis (SID-GBS): a double-blind, randomised, placebo-controlled trial. The Lancet Neurology, 20(4), 275-283.)

The simulated data set (wide/long format) has the following variables:

- Patient identifier: id
- Outcome: GBS-DS, a seven scale ordinal neuro-functionality score, but combined score 0 with 1 and score 6 with 5 due to small group sizes. Repeated measurements at all visits, no missing data
- Treatment assignment: trt, 1:1 equal randomization
- Covariate: age, continuous from a normal distribution with mean 60 and sd 10
- Covariate: pre_diarrhea: binary, yes(1)/no(0), prevalence is 40%

```r
# set simulation parameters
# sample size (two arms), 1:1 randomization
N <- 100
# base category probabilities when all covariates are zero
baseprobs <- c(0.02,0.06,0.11,0.12,0.47,0.21,0.01)
# number of visits, unit is week
visits <- paste0("week",c(0,1,2,4,8,12,26)) # baseline is visit = 0
# simulate data from a proportional odds model
# because lower GBS-DS scores are better
# and we model the logit of P(Y >= j)
# so negative coefficients means beneficial effect
# (OR < 1 of a worse GBS-DS score)
# positive time trend towards higher score (self-healing)
time_effects <- -0.05*c(1,2,4,8,12,26)
# covariate effects estimated from SID trial
covs_effects <- c("age"=0.005,"pre_diarrhea"= -0.23)
# time by treatment interaction
time_trt_effects <- -0.05*c(1,2,4,8,12,26)
# correlation structure, the simstudy package generates correlated values from
# the logistic distribution using a standard normal copula-like approach
# with supplied correlation matrix
rho <- 0.6 ; corstr <- "ar1"
corMatrix <- lwo::gen_corMatrix(n_visits = length(visits),rho=rho,corstr = corstr)

# get the simulated data set
set.seed(86)
dat <-  lwo::gen_data(N = N,
                  baseprobs = baseprobs,
                  covs_effects = covs_effects,
                  time_effects = time_effects,
                  time_trt_effects = time_trt_effects,
                  visits = visits,
                  corMatrix = corMatrix,
                  extractTime = TRUE)
```

```
dat_wide <- dat$wide_format
dat_long <- dat$long_format

# change variable names to match the SID trial data set
colnames(dat_wide)[which(colnames(dat_wide)=="id")] <- "patient_ID"
colnames(dat_wide)[which(colnames(dat_wide)=="trt")] <- "treat_ITT"
colnames(dat_wide)[5:11] <- paste0("GBS_DS_week",c(0,1,2,4,8,12,26))
colnames(dat_long)[which(colnames(dat_long)=="id")] <- "patient_ID"
colnames(dat_long)[which(colnames(dat_long)=="time")] <- "week"
colnames(dat_long)[which(colnames(dat_long)=="trt")] <- "treat_ITT"
colnames(dat_long)[which(colnames(dat_long)=="score")] <- "GBS_DS"

# change GBS-DS range from 1-7 to 0-6 scale
dat_wide[,
         paste0("GBS_DS_week",
                c(0,1,2,4,8,12,26))] <- dat_wide[,
                                                 paste0("GBS_DS_week",
                                                        c(0,1,2,4,8,12,26))] - 1
dat_long$GBS_DS <- dat_long$GBS_DS - 1
```

Exploratory analysis

Proportion of categories over time

```
# The frequency distribution of ordinal categories over time
tab <- as.data.frame.matrix(table("week"=dat_long$week,
                                  "GBS-DS"=dat_long$GBS_DS))
colnames(tab) <- paste0("GBS-DS-", sort(unique(dat_long$GBS_DS)))
rownames(tab) <- paste0("week",c(0,1,2,4,8,12,26))

# visualize observed proportions of each category over time by treatment group
cat_prop <- plyr::count(dat_long,c("treat_ITT","week","GBS_DS"))
cat_prop$treat_ITT <- factor(cat_prop$treat_ITT,levels = c(0,1),
                             labels = c("Control","Intervention"))
cat_prop |>
  ggplot(aes(x=factor(week),y=freq,fill=factor(GBS_DS)))+
  facet_wrap(~treat_ITT)+
  geom_bar(position = "fill",stat = "identity")+
  labs(
       x="Week",
```

```
      y="Proportion",
      fill="GBS-DS")+
  theme_minimal()+
  scale_fill_manual(values = blues)+
  theme(
    strip.text.x = element_text(size=12)
  )
```

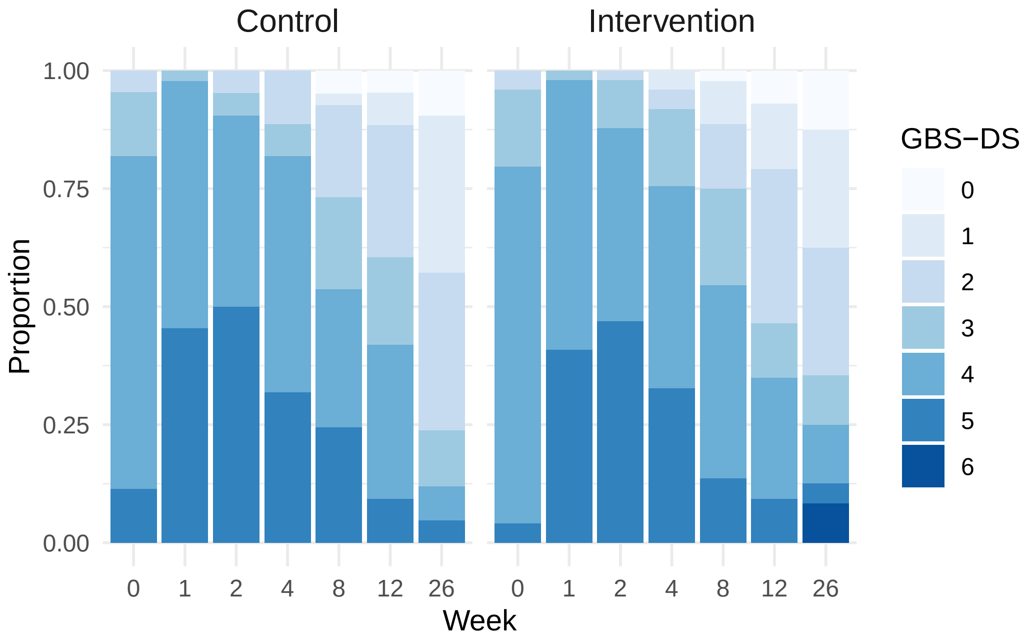

Figure 1: Stacked Bar Chart of Observed Proportion of Each GBS-DS Score over Time

Empirical cumulative probability of each category

```
# get empirical CDF for each category over time
dat_long |>
  mutate(GBS_DS = as.factor(GBS_DS),
         treat = as.factor(treat_ITT)) |>
  group_by(week,treat,GBS_DS) |>
  dplyr::count() |>
  group_by(week,treat) |>
  dplyr::mutate(count=sum(n)) |>
  dplyr::mutate(cumprob=n/count) |>
```

```r
ggplot() +
aes(x = week, y = cumprob, color=GBS_DS, linetype=treat) +
geom_line(lwd=0.8) +
geom_point(alpha=0.6, size=0.5) +
scale_x_continuous(breaks = c(0,1,2,4,8,12,26)) +
scale_y_continuous(breaks = seq(0, 1, by=0.1)) +
coord_cartesian(ylim = c(0,0.8)) +
labs(x = "Study week",
     y = "Proportion",
     color =  "GBS-DS",
     linetype = "Treatment") +
scale_color_manual(values = blues)+
theme_minimal()+
scale_linetype_manual(values = c(1,2),
                                 labels=c("Intervention","Control"))
```

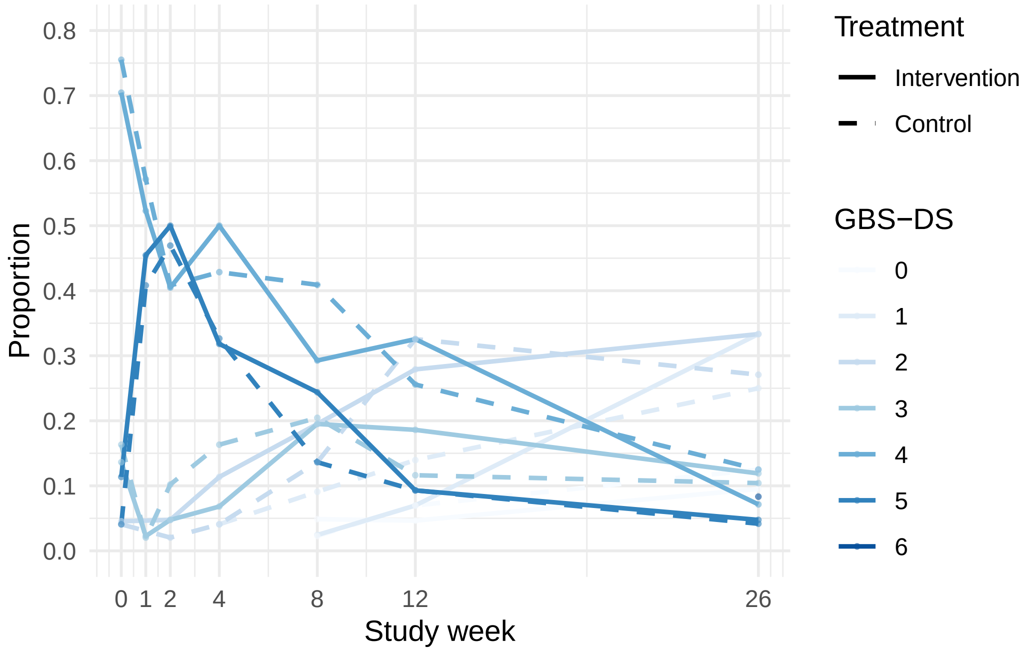

Figure 2: Line Chart of Observed Proportion of Each GBS-DS Score over Time

Individual horizontal line plot

```
# order patients for better illustration
ID_order0 <- dat_wide |>
  filter(treat_ITT==0) |>
 # arrange(visit1,visit2,visit3,visit4,visit5,visit6,visit7) |>
  arrange(GBS_DS_week0,GBS_DS_week1,GBS_DS_week2,GBS_DS_week4,
          GBS_DS_week8,GBS_DS_week12,GBS_DS_week26) |>
  select(patient_ID) |>
  unlist() |>
  as.vector()
ID_order1 <- dat_wide |>
  filter(treat_ITT==1) |>
  arrange(GBS_DS_week0,GBS_DS_week1,GBS_DS_week2,GBS_DS_week4,
          GBS_DS_week8,GBS_DS_week12,GBS_DS_week26) |>
  select(patient_ID) |>
  unlist() |>
  as.vector()
dat_long_ordered <- bind_rows(sapply(c(ID_order0,ID_order1), function(id) {
  df <- dat_long[which(dat_long$patient_ID==id),]
  if(id %in% ID_order0)
  {
    df$order <- rep(which(ID_order0==id),nrow(df))
  } else
  {
    df$order <- rep(which(ID_order1==id),nrow(df))
  }
  return(df)
},simplify = FALSE))

dat_long_ordered <- dat_long_ordered |>
  mutate(id = as.factor(order),treat_ITT=factor(treat_ITT,
                                                 levels=c(0,1),
                                                 labels=c("Control","Intervention")))


p0 <- ggplot(subset(dat_long_ordered,treat_ITT=="Control")) +
  aes(y=id, x=factor(week)) +
  geom_tile(mapping = aes(fill = as.factor(GBS_DS)),
            width=0.95, height=1) +
  scale_fill_manual(values = blues) +
```

```
  expand_limits(fill = c(as.character(0:6))) +
  labs(x = "Study week",
       y = "One patient per line",
       fill = "GBS-DS",
       title = "Control")+
  theme(
    axis.text.y = element_blank(),
    axis.ticks.y = element_blank(),
     plot.title = element_text(size=12)
  )
p1 <- ggplot(subset(dat_long_ordered,treat_ITT=="Intervention")) +
  aes(y=id, x=factor(week)) +
  geom_tile(mapping = aes(fill = as.factor(GBS_DS)),
            width=0.95, height=1) +
  scale_fill_manual(values = blues) +
  expand_limits(fill = c(as.character(0:6))) +
  labs( x="",
      y = "One patient per line",
       fill = "GBS-DS",
      title = "Intervention")+
  theme(
    axis.text.y = element_blank(),
    axis.ticks.y = element_blank(),
    plot.title = element_text(size=12)
  )
ggarrange(p1,p0,ncol=1,nrow=2,common.legend = TRUE,
          legend = "right")
```

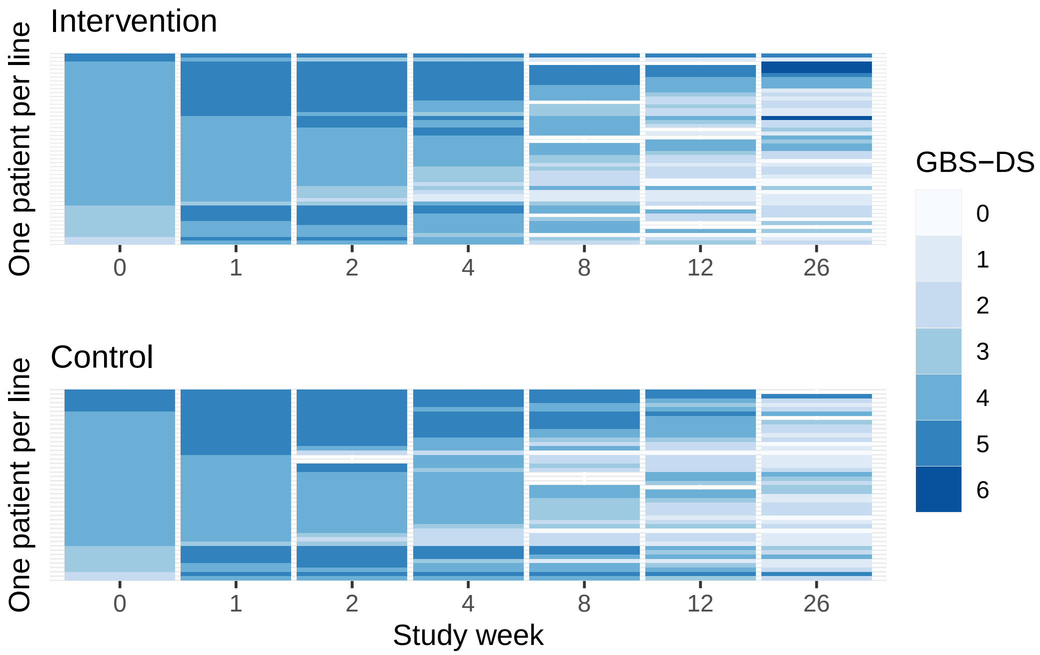

Figure 3: Individual trajectory plot by treatment group. Ordered by GBS-DS severity chronologically.

Separate cross-sectional win odds analysis

For later comparison, we calculate the win odds at all available visits of GBS-DS (primary endpoint) from single-time probabilistic index model. We calculate both the unadjusted win odds and the ones adjusted for age, preceding diarrhea and baseline GBS-DS score.

```
covs <- paste0("GBS_DS_week",c(1,2,4,8,12,26))
# unadjusted win odds
wo.cs.unadj <- sapply(covs, function(cov)
  {
  data <- dat_wide
  # by default PIM thinks larger outcome is a win,
  # to accommodate that smaller GBS-DS is better
  # we reverse the outcome
  data$outcome <- 6 - data[[cov]]
  formula <- as.formula("outcome~treat_ITT")
  mod.adj <- pim(formula = formula,data = data[!is.na(data$outcome),])
  wo.adj <- c(exp(confint(mod.adj)["treat_ITT",])[1],
              exp(mod.adj@coef["treat_ITT"]),
```

```
                exp(confint(mod.adj)["treat_ITT",])[2])
  names(wo.adj) <- c("lower.CI","estimate","upper.CI")
  return(wo.adj)
}) |>
  t() |>
  as.data.frame()
wo.cs.adj <- sapply(covs, function(cov)
  {
  data <- dat_wide
  # by default PIM thinks larger outcome is a win,
  # to accommodate that smaller GBS-DS is better
  # we reverse the outcome
  data$outcome <- 6 - data[[cov]]
  formula <- as.formula("outcome~treat_ITT+age+pre_diarrhea+GBS_DS_week0")
  mod.adj <- pim(formula = formula,data = data[!is.na(data$outcome),])
  wo.adj <- c(exp(confint(mod.adj)["treat_ITT",])[1],
              exp(mod.adj@coef["treat_ITT"]),
              exp(confint(mod.adj)["treat_ITT",])[2])
  names(wo.adj) <- c("lower.CI","estimate","upper.CI")
  return(wo.adj)
}) |>
  t() |>
  as.data.frame()
```

Longitudinal win odds analysis

In the longitudinal analysis, we use the same baseline variables as the cross-sectional analysis: age, preceding diarrhea and baseline GBS-DS score.

Analysis 1: Average win odds over time

We first estimate an average treatment effect over time. Namely, we do not include time-by-treatment interaction terms and estimate one win odds for the treatment.

We need to supply the lwo() function with:

- formula: GBS_DS ~ treat_ITT + …
- data: the data frame in long format, the function internally convert individual-level data to pair-level data
- id.var: character string of cluster id

- visit.var: variable name that distinguishes the visit
- time.vars: Optional, needed if time-varying effect is modeled. Should be a vector of variables names that represent time. This is needed because PIM does not have intercept, so there will not be main effects of time in the model. Time variables will not be converted to pair level. But it is valid to include interaction terms between time variables and some covariates (such as treatment) to allow the covariate effect change over time
- corstr: working correlation structure among repeated measurements. The function internally calls geese.fit() for parameter estimation. So the syntax for supplying working correlation structure is the same as geeglm()
- larger: whether larger values of the ordinal category means better outcome conditions (a win). Default is TRUE. But here we need to change to FALSE because for GBS-DS smaller values mean better outcome

```
# construct baseline GBS DS as a predictor
dat_long$GBS_DS_baseline <- rep(dat_wide$GBS_DS_week0,
                                as.vector(table(dat_long$patient_ID)))
# delete baseline as an outcome
dat_long <- dat_long[dat_long$week>0,]
# model average win odds, unadjusted
mod.lwo.avg.unadj <- lwo::lwo(
  GBS_DS ~ treat_ITT,
  data = dat_long,
  id.var = "patient_ID",
  visit.var = "week",
  corstr = "ar1",
  larger = FALSE
)
summary(mod.lwo.avg.unadj)
```

```
Call:
lwo::lwo(formula = GBS_DS ~ treat_ITT, data = dat_long, id.var = "patient_ID",
    visit.var = "week", larger = FALSE, corstr = "ar1")

Coefficients:
          Estimate Std.err  Wald Pr(>|W|)
treat_ITT  0.01058 0.17403 0.004    0.952

Temporal Correlation Structure: ar1
```

```
# model average win odds, adjusted
mod.lwo.avg.adj <-  lwo::lwo(
  GBS_DS ~ treat_ITT + age + pre_diarrhea+ GBS_DS_baseline,
  data = dat_long,
  id.var = "patient_ID",
  visit.var = "week",
  corstr = "ar1",
  larger = FALSE
)
summary(mod.lwo.avg.adj)
```

```
Call:
lwo::lwo(formula = GBS_DS ~ treat_ITT + age + pre_diarrhea +
    GBS_DS_baseline, data = dat_long, id.var = "patient_ID",
    visit.var = "week", larger = FALSE, corstr = "ar1")

Coefficients:
                 Estimate   Std.err  Wald Pr(>|W|)
treat_ITT        0.044909  0.184327 0.059    0.808
age             -0.003002  0.004911 0.374    0.541
pre_diarrhea    -0.036017  0.189568 0.036    0.849
GBS_DS_baseline -0.006528  0.148848 0.002    0.965

Temporal Correlation Structure: ar1
```

Analysis 2: Response profile modelling (time as a categorical covariate)

In this analysis, we use an unstructured model for the probabilistic index. Namely, we don't assume a parametric form for the treatment effect trajectory, instead, we model time categorically.

```
# model time categorically, create dummies, use week 1 as the reference
dat_long <- dummy_cols(dat_long,
                  select_columns = "week",
                  remove_first_dummy = TRUE)
# model time-varying win odds, unadjusted
mod.lwo.timevarying.unadj <- lwo::lwo(
  GBS_DS ~ treat_ITT +
    treat_ITT:week_2+
    treat_ITT:week_4+
```

```r
    treat_ITT:week_8+
    treat_ITT:week_12+
    treat_ITT:week_26,
  data = dat_long,
  id.var = "patient_ID",
  visit.var = "week",
  time.vars = paste0("week_",c(2,4,8,12,26)),
  corstr = "ar1",
  larger = FALSE
)
summary(mod.lwo.timevarying.unadj)
```

```
Call:
lwo::lwo(formula = GBS_DS ~ treat_ITT + treat_ITT:week_2 + treat_ITT:week_4 +
    treat_ITT:week_8 + treat_ITT:week_12 + treat_ITT:week_26,
    data = dat_long, id.var = "patient_ID", visit.var = "week",
    time.vars = paste0("week_", c(2, 4, 8, 12, 26)), larger = FALSE,
    corstr = "ar1")

Coefficients:
                   Estimate   Std.err  Wald Pr(>|W|)
treat_ITT          0.087334  0.210298 0.172    0.678
treat_ITT:week_2  -0.025319  0.152021 0.028    0.868
treat_ITT:week_4  -0.005944  0.187831 0.001    0.975
treat_ITT:week_8   0.031869  0.221324 0.021    0.886
treat_ITT:week_12  0.123701  0.249069 0.247    0.619
treat_ITT:week_26 -0.269255  0.278323 0.936    0.333

Temporal Correlation Structure: ar1
```

```r
# model time-varying win odds, adjusted
mod.lwo.timevarying.adj <- lwo::lwo(
  GBS_DS ~ treat_ITT + age + pre_diarrhea + GBS_DS_baseline+
    treat_ITT:week_2+
    treat_ITT:week_4+
    treat_ITT:week_8+
    treat_ITT:week_12+
    treat_ITT:week_26,
  data = dat_long,
  id.var = "patient_ID",
  visit.var = "week",
```

```r
  time.vars = paste0("week_",c(2,4,8,12,26)),
  corstr = "ar1",
  larger = FALSE
)
summary(mod.lwo.timevarying.adj)
```

```
Call:
lwo::lwo(formula = GBS_DS ~ treat_ITT + age + pre_diarrhea +
    GBS_DS_baseline + treat_ITT:week_2 + treat_ITT:week_4 + treat_ITT:week_8 +
    treat_ITT:week_12 + treat_ITT:week_26, data = dat_long, id.var = "patient_ID",
    visit.var = "week", time.vars = paste0("week_", c(2, 4, 8,
        12, 26)), larger = FALSE, corstr = "ar1")

Coefficients:
                   Estimate   Std.err  Wald Pr(>|W|)
treat_ITT          0.122464  0.221168 0.307    0.580
age               -0.002937  0.004903 0.359    0.549
pre_diarrhea      -0.038103  0.189268 0.041    0.840
GBS_DS_baseline   -0.003854  0.148421 0.001    0.979
treat_ITT:week_2  -0.024919  0.152257 0.027    0.870
treat_ITT:week_4  -0.005071  0.188143 0.001    0.978
treat_ITT:week_8   0.033215  0.221764 0.022    0.881
treat_ITT:week_12  0.126899  0.249299 0.259    0.611
treat_ITT:week_26 -0.266683  0.278516 0.917    0.338

Temporal Correlation Structure: ar1
```

Result visualization

To better examine the estimated treatment effect (quantified by win odds) trajectory, we use the predict() function to get estimated win odds at each visit. For analysis 1, we get the same estimated win odds for all visits because we model only one averaged effect.

```r
# construct a data frame (long format) with one treated patient and one
# control patient, keep other covariates the same
newdata <- dat_long[dat_long$patient_ID==1,]
nvisits <- nrow(newdata)
newdata <- rbind(newdata,newdata)
newdata$patient_ID[(nvisits+1):(2*nvisits)] <- 2
newdata$treat_ITT <- rep(c(0,1),each=nvisits)
```

```
est.wo.avg.unadj <- predict(object=mod.lwo.avg.unadj,
                            newdata = newdata,
                            id.var="patient_ID",
                            visit.var="week",
                            type="link",conf.int = TRUE) |>
  exp() |>
  data.frame()
est.wo.avg.adj <- predict(mod.lwo.avg.adj,newdata,
                   id.var="patient_ID",
                   visit.var="week",
                   type="link",conf.int = TRUE) |>
  exp() |>
  data.frame()

est.wo.timevarying.unadj <- predict(mod.lwo.timevarying.unadj,newdata,
                   id.var="patient_ID",
                   visit.var="week",
                   time.vars = paste0("week_",c(2,4,8,12,26)),
                   type="link",conf.int = TRUE) |>
  exp() |>
  data.frame()
est.wo.timevarying.adj <- predict(mod.lwo.timevarying.adj,newdata,
                   id.var="patient_ID",
                   visit.var="week",
                   time.vars = paste0("week_",c(2,4,8,12,26)),
                   type="link",conf.int = TRUE) |>
  exp() |>
  data.frame()
wo_df <- rbind(wo.cs.unadj,
               wo.cs.adj,
               est.wo.avg.unadj,
               est.wo.avg.adj,
               est.wo.timevarying.unadj,
               est.wo.timevarying.adj)
wo_df$Type <- rep(1:6,each = 6)
wo_df$Type <- factor(wo_df$Type,levels = 1:6,
                     labels = c("cross-sectional, unadjusted",
                    "cross-sectional, adjusted",
                    "longitudinal, average, unadjusted",
                     "longitudinal, average, adjusted",
                     "longitudinal, time-varying, unadjusted",
```

```r
                    "longitudinal, time-varying, adjusted"))
wo_df$Week <- rep(c(1,2,4,8,12,26),6)

ggplot(wo_df,aes(x=factor(Week),y=estimate,color=Type,group=Type))+
  geom_point(position=position_dodge(width=0.4))+
  theme_minimal()+
  geom_hline(yintercept = 1,color="darkgrey",linetype=2)+
  geom_errorbar(aes(ymin=lower.CI,ymax=upper.CI),width=0.5,
                position=position_dodge(width=0.4))+
  ggsci::scale_color_nejm()+
  labs(x="Week",y="Win odds for the treatment")+
  theme(
    legend.position = "bottom"
  )
```

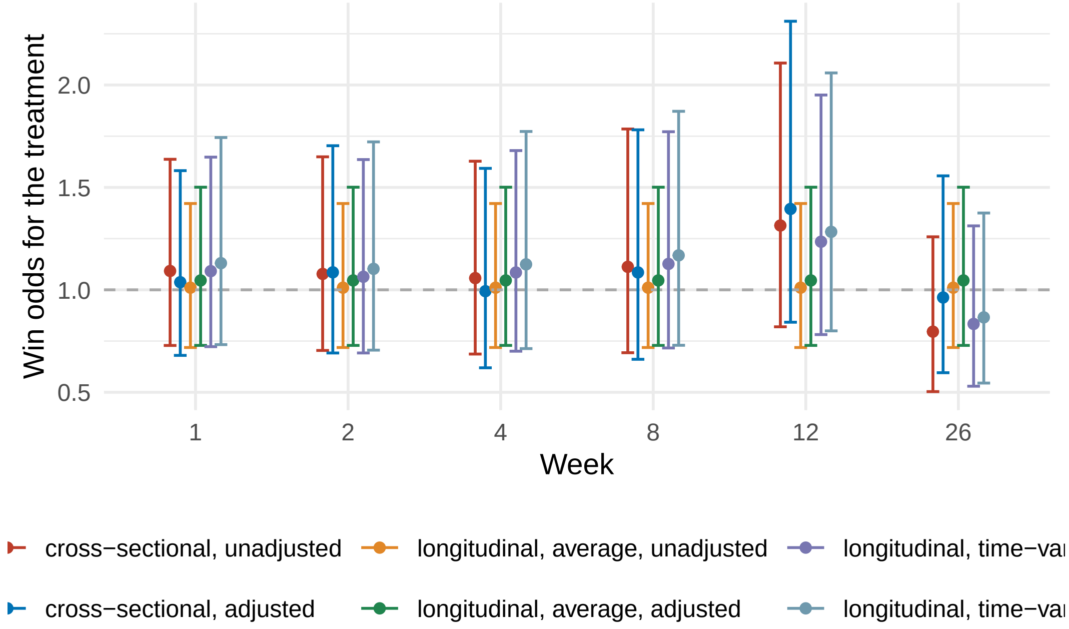

Analysis 3: model treatment effect trajectory through spline

From exploratory data analysis, we see a curved shape of individual patient trajectories over time, and also we see the distribution of the GBS-DS kind of shifted up first (week 0 to week 2) and then went down. So we model the treatment effect trajectory in the natural cubic spline form with one knot at week 2 (degree of freedom = 2, allows the curve to "bend" once).

```r
mod.lwo.spline <- lwo(
  GBS_DS ~ treat_ITT + age + pre_diarrhea + GBS_DS_baseline+
    treat_ITT:ns(week,knots = 2),
  data = dat_long,
  id.var = "patient_ID",
  visit.var = "week",
  time.vars = "week",
  corstr = "ar1",
  larger = FALSE
)
summary(mod.lwo.spline)
```

```
Call:
lwo(formula = GBS_DS ~ treat_ITT + age + pre_diarrhea + GBS_DS_baseline +
    treat_ITT:ns(week, knots = 2), data = dat_long, id.var = "patient_ID",
    visit.var = "week", time.vars = "week", larger = FALSE, corstr = "ar1")

Coefficients:
                                Estimate   Std.err  Wald Pr(>|W|)
treat_ITT                       0.091425  0.215314 0.180    0.671
age                            -0.002913  0.004901 0.353    0.552
pre_diarrhea                   -0.037957  0.189379 0.040    0.841
GBS_DS_baseline                -0.003689  0.148396 0.001    0.980
treat_ITT:ns(week, knots = 2)1  0.209110  0.460563 0.206    0.650
treat_ITT:ns(week, knots = 2)2 -0.327576  0.204585 2.564    0.109

Temporal Correlation Structure: ar1
```

Result visualization

To get win odds for a sequence of time points (week 1 till week 26), construct a new data frame and use the predict() function.

```r
# we want to get win odds from week 1 till week 26, conditional on that
# the pairs have the same age and same preceding diarrhea status and same baseline GBS-DS
# so set age and pre_diarrhea and GBS_DS_baseline to 0
nvisits <- 26
newdata <- data.frame(
  patient_ID = rep(c(1,2),each=nvisits),
  week = rep(1:nvisits,2),
```

```
  treat_ITT = rep(c(0,1),each=nvisits),
  age = rep(60,nvisits*2),
  pre_diarrhea = rep(0,2*nvisits),
  GBS_DS_baseline = rep(4,2*nvisits)
)
pred.lp <- predict(mod.lwo.spline,newdata,
                   id.var="patient_ID",
                   visit.var="week",
                    time.vars = "week",
                   type="link",conf.int = TRUE)
pred.wo <- exp(pred.lp)
# odds scale
wo_df_spline <- data.frame(cbind(week=1:26,pred.wo))
ggplot(wo_df_spline,aes(x=week,y=estimate))+
  geom_line()+
  geom_point()+
  theme_minimal()+
  scale_x_continuous(breaks = 1:26)+
  geom_hline(yintercept = 1,color="darkgrey",linetype=2)+
  geom_errorbar(aes(ymin=lower.CI,ymax=upper.CI),width=.3,color="#4d79a6")+
  labs(x="Week",y="Win odds for the treatment")
```

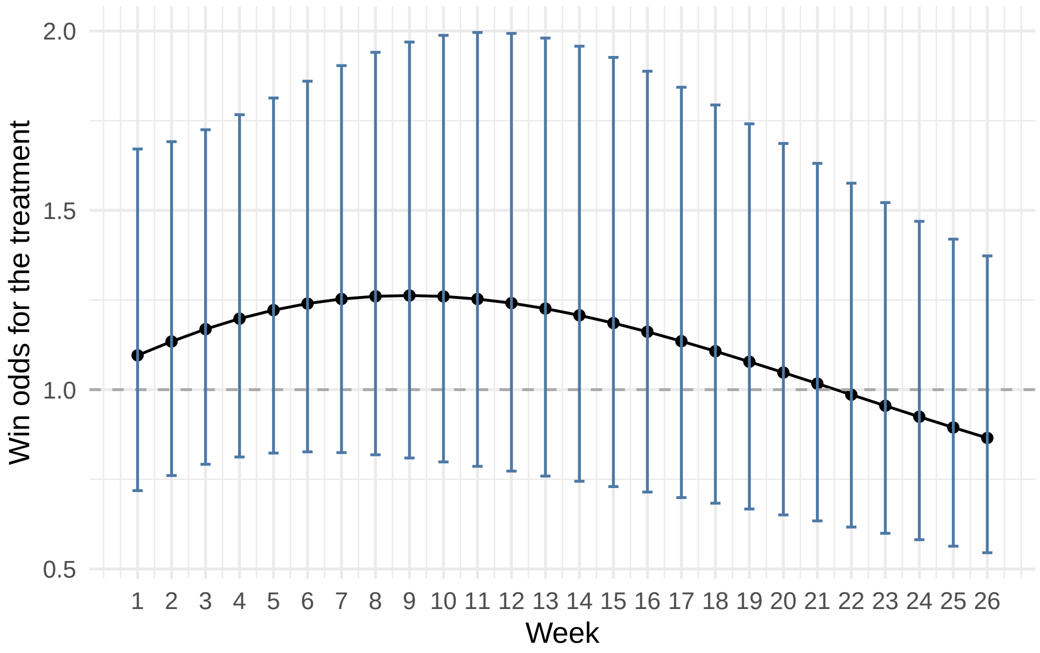

Supplement: Supplementary file 2 — Appendix S2: sim70536‐sup‐0002‐AppendixS2.pdf. [file SIM-45-0-s002.pdf]
